# Complete mitochondrial genome of the snowy sheathbill (*Chionis albus*, Charadriiformes: chionidae) and its phylogenetic implications

**DOI:** 10.1080/23802359.2026.2658389

**Published:** 2026-04-18

**Authors:** Jihee Kim, Jong-U Kim, Jeong-Hoon Kim

**Affiliations:** Division of Life Sciences, Korea Polar Research Institute, Incheon, South Korea

**Keywords:** Antarctica, *Chionis albus*, mitogenome, phylogenetic analysis, snowy sheathbill

## Abstract

The snowy sheathbill (*Chionis albus* (Gmelin, 1789)) is a coastal Antarctic bird that breeds on Antarctic and sub-Antarctic islands. In this study, we report the first complete mitochondrial genome sequence of *C. albus*. The mitogenome is circular, 19,116 bp in length, and contains 13 protein-coding genes (PCGs), including an *ND3* gene with an extra nucleotide, 22 transfer RNAs (tRNAs), and two ribosomal RNAs (rRNAs). Notably, a duplicated region of ∼2.3 kb includes *ND6* and two tRNAs. The overall nucleotide composition is 30.29% A, 30.37% C, 14.56% G, and 24.78% T, with a GC content of 44.93%. Phylogenetic analysis based on all 13 PCGs placed *C. albus* firmly within Charadriiformes and clustered with *C. minor* and, more distantly, with *Burhinus bistriatus*. This newly characterized mitogenome provides valuable resources for future studies on the phylogeny, molecular evolution, and biogeography of Charadriiformes and the family Chionidae.

## Introduction

**Figure 1.**
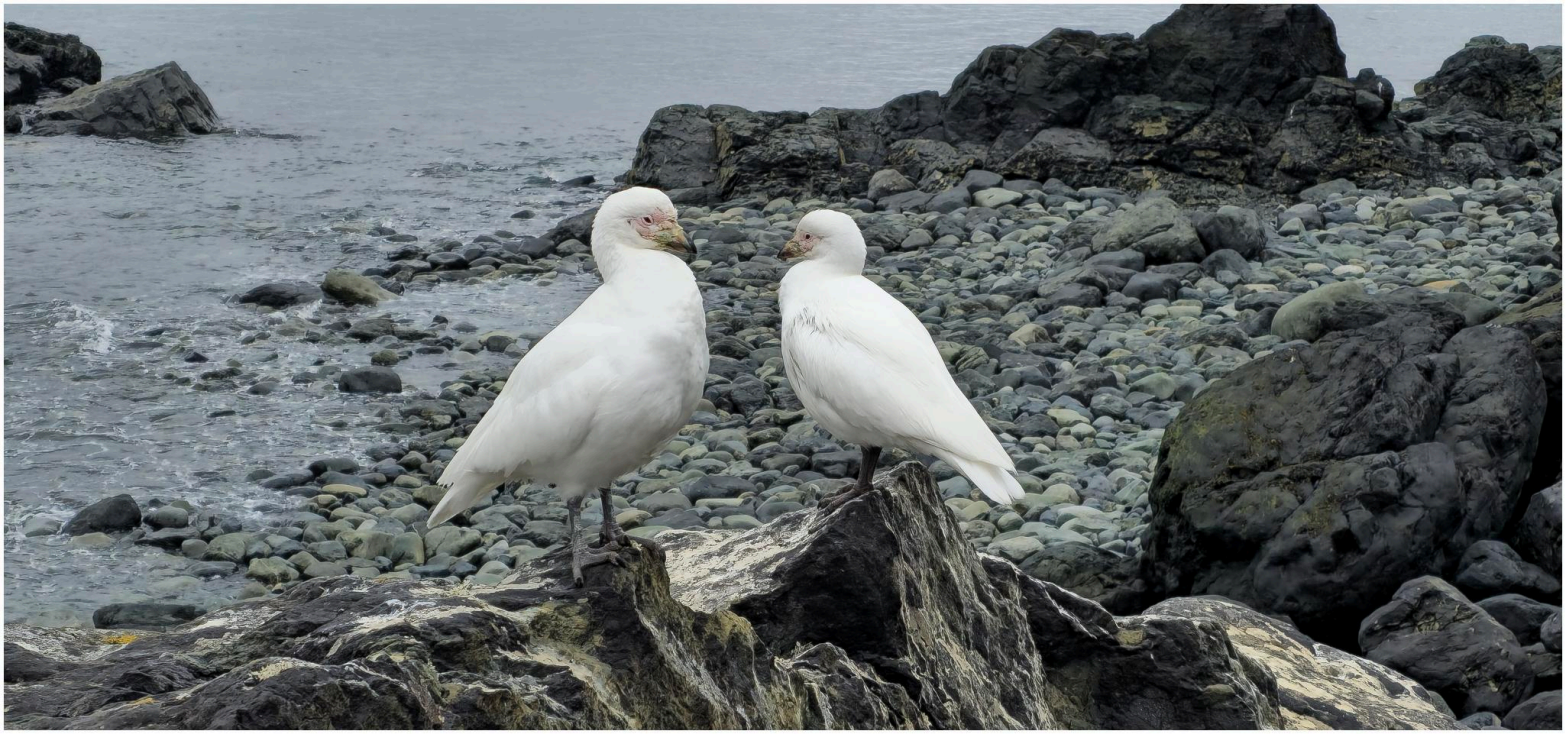
Species reference image of *Chionis albus* at the Barton Peninsula, showing pale bill and bare loral region that distinguish it from *Chionis minor*. The species identification was confirmed in the field by Dr. Jeong-hoon Kim, who also took the photograph.

*Chionis albus* (Gmelin, 1789), known as the snowy sheathbill, is one of the two extant species in the family Chionidae, order Charadriiformes (Figure 1). The family Chionidae comprises a single genus, *Chionis*, including *C. albus* and *C. minor* (Hartlaub, 1841) (Lin et al. 2018; Hernandez et al. 2021). Although both species share a white plumage, they differ in bill and facial morphology (Shirihai 2019). *Chionis albus* is a coastal Antarctic species that breeds during the austral summer on Antarctic and sub-Antarctic islands as well as along the Antarctic Peninsula (Jones 1963; Harris et al. 2015). The species is categorized as ‘Least Concern’ (IUCN 2024), with approximately 10,000 breeding pairs (Shirihai 2019). Mitochondrial genome data are widely used in studies on molecular evolution and taxonomic clarification (Miya and Nishida 2015; Kim and Kim 2025). Although previous phylogenetic studies have included *C. albus* (Baker et al. 2012; Reddy et al. 2017), its complete mitochondrial genome has not yet been reported. As part of an ongoing research program that focuses on Antarctic birds (Han et al. 2016, Jung et al. 2019, Kim and Kim 2021, 2023, 2025), we characterized the complete mitochondrial genome of *C. albus* and inferred its phylogenetic relationships with related species using mitochondrial protein-coding genes (PCGs). These data will improve understanding of the evolutionary history of this species and provide a foundation for future phylogenetic studies.

## Materials and methods

An adult *C. albus* was manually captured at its nest at the Barton Peninsula, Antarctica (62°1′’0.36″S, 58°46′56.20″W), on January 18, 2015. The species identification was confirmed by Dr. Jeong-Hoon Kim prior to blood sampling. Approximately 100 µl of blood was collected from a major wing vein using disposable syringes within 10 min of capture. No anesthesia was used; instead, the bird was gently handled during the brief sampling procedure to minimize stress. The blood sample (voucher ID: PFS2) was preserved in a frozen state at the Korea Polar Research Institute, Incheon, South Korea (https://www.kopri.re.kr, Dr. Jeong-Hoon Kim, jhkim94@kopri.re.kr) until genomic DNA extraction and sequencing in 2018. Total genomic DNA was extracted from the blood sample using a DNeasy Blood and Tissue kit (Qiagen, Hilden, Germany) following the manufacturer’s instructions. The complete mitochondrial genome of *C. albus* was sequenced and analyzed following the protocol described by Kim and Kim (2021). Briefly, a TruSeq DNA PCR-free library was prepared, and 151-bp paired-end sequencing was performed on an Illumina HiSeq 2500 platform by Phyzen (Seongnam, South Korea). Raw Illumina reads were quality-trimmed and assembled into the complete mitochondrial genome using the CLC Assembly Cell package v4.2.1 (QIAGEN, Denmark). Genes were annotated with GeSeq (Tillich et al. 2017) and refined using Artemis (Rutherford et al. 2000). A circular genome map was generated with PMG map (Zhang et al. 2024). Genome completeness was assessed by mapping raw reads back to the assembled genome and inspecting the depth-of-coverage profiles. Phylogenetic inference of *C. albus* was conducted using orthologous mitochondrial PCGs together with 17 avian species: 16 Charadriiformes (six Charadrii, three Scolopaci, and seven Lari) and one additional species, *Grus grus* (Gruiformes) as the outgroup. Alignments were generated using MAFFT v7.526, and maximum-likelihood phylogenies were reconstructed in MEGA11 (Tamura et al. 2021) using the GTR+G + I model with 1,000 bootstrap replicates.

## Results

**Figure 2.**
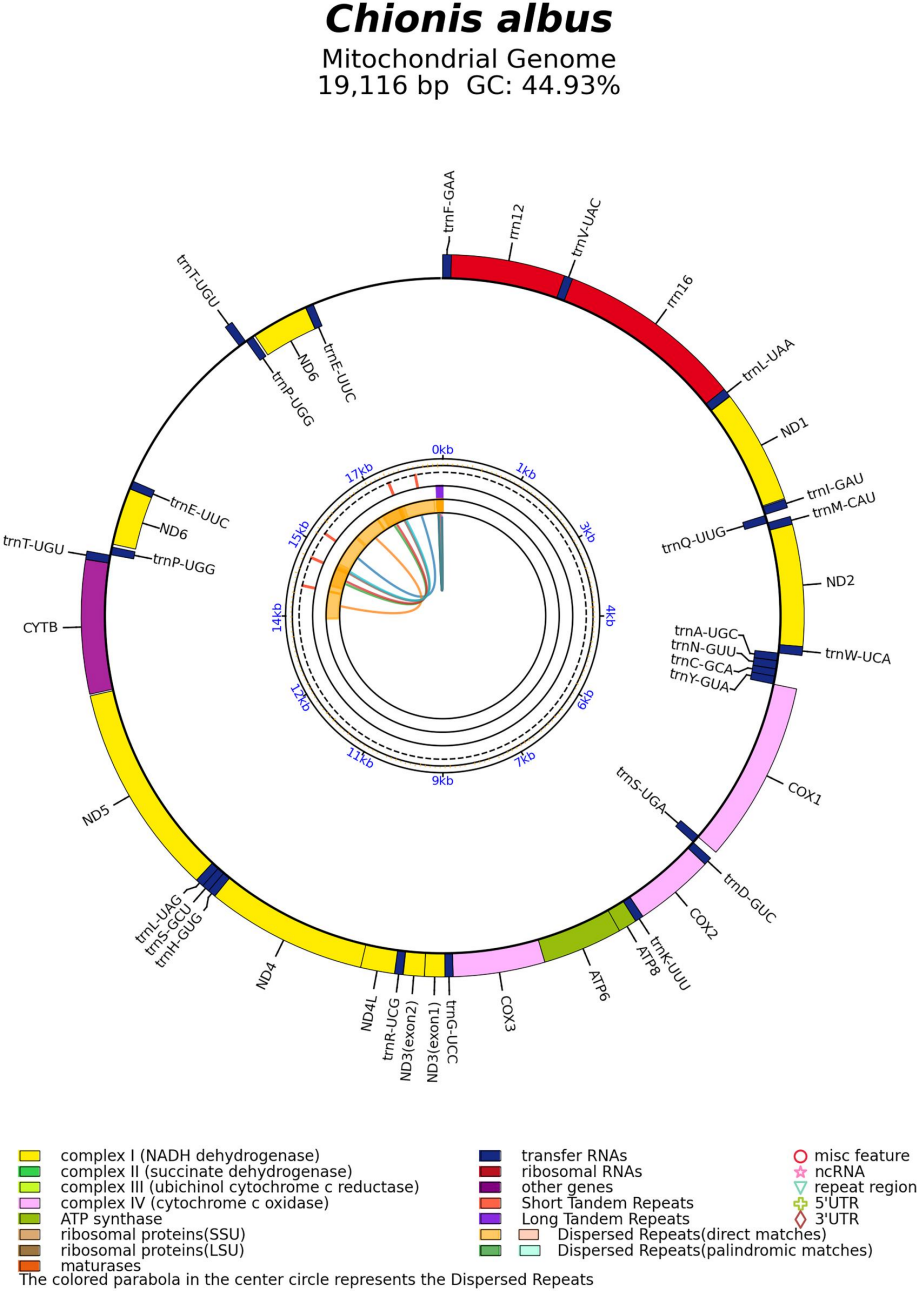
Circular map of *Chionis albus* mitochondrial genome, containing 13 protein-coding genes, 22 tRNA genes, and two rRNA genes. Genes encoded on the heavy strand are displayed on the outside of the circle, whereas those encoded on the light strand are shown inside.

**Figure 3.**
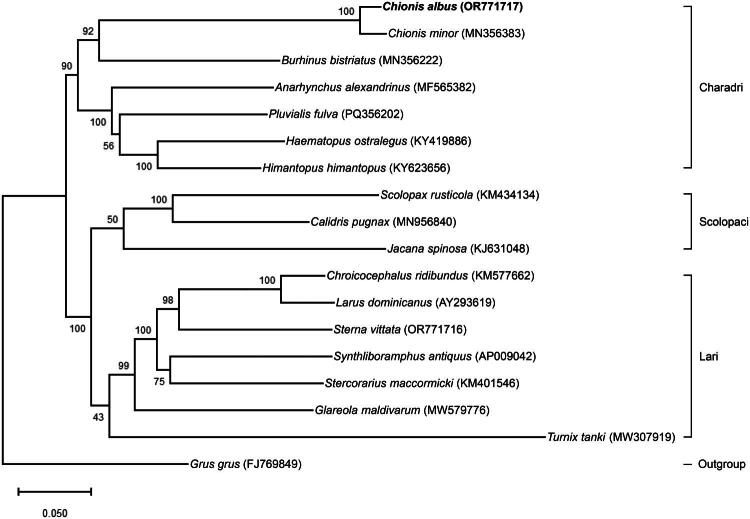
Maximum-likelihood (ML) phylogenetic tree of *Chionis albus*, related charadriiformes, and the outgroup taxon based on mitochondrial PCGs. Numbers on the branches indicate the ML bootstrap percentages of 1,000 replicates. The following sequences with GeneBank accession numbers were used: *Chionis albus* OR771717 (this study); *Chionis minor* MN356383 (Feng et al. 2020; partial mitogenome); *Burhinus bistriatus* MN356222 (Feng et al. 2020); *Anarhynchus alexandrinus* MN356435 (Feng et al. 2020); *Pluvialis fulva* PQ356202 (Sun et al. 2024); *Haematopus ostralegus* KY419886 (Lee et al. 2017); *Himantopus himantopus* KY623656 (Yang et al. 2017); *Scolopax rusticola* KM434134 (Yu et al. 2016); *Calidris pugnax* MN956840 (Chen et al. 2020); *Jacana spinosa* KJ631048 (Miller et al. 2016); *Chroicocephalus ridibundus* KM577662 (Dong et al. 2016); *Larus dominicanus* AY293619 (Slack et al. 2007); *Sterna vittata* OR771716 (Kim and Kim 2023); *Synthliboramphus antiquus* AP009042 (Yamamoto et al. 2005); *Atercorarius maccormicki* KM401546 (Han et al. 2016); *Glareola maldivarum* MW579776 (Peng et al. 2022); *Turnix tanki* MW307919 (Gou et al. 2021) and *Grus grus* FJ769849 (O’Brien et al. 2025).

The complete mitochondrial genome of *C. albus* (OR771717) has a closed-circular form with a total length of 19,116 bp (Figure 2). Within the genome, two duplicated regions are present (Supplementary Figure S1), spanning ∼2.3 kb. In addition to these duplications, the genome contains 22 transfer RNAs (tRNAs), two ribosomal RNAs (rRNAs), and 13 PCGs. Among the PCGs, *ND3* contained an extra nucleotide at position 9713 bp, corresponding to position 174 of the *ND3* gene (Supplementary Figure S2). The overall nucleotide base composition is 30.29% (A), 30.37% (C), 14.56% (G), and 24.78% (T), with a GC content of 44.93%. The heavy strands encode 28 genes, including 12 PCGs, two rRNA genes, and 14 tRNA genes. In contrast, the light strands encode nine genes, including one PCG (*ND6*) and eight tRNA genes. The 13 PCGs of *C. albus* encode 3,793 amino acids. Most PCGs are initiated with ATG, except *COX1* and *ND2* (GTG) and *ND3* (ATC). Stop codons were predominantly TAA, with occasional TAG, AGA, or AGG. *COX3* and *ND4* had incomplete stop codons (T(AA) or TA(A)), completed by 3′ A residues. This was followed by TAG in *ND2* and *ND6*, AGA in *ND1* and *ND5*, and AGG in *COX1*. A phylogenetic tree was constructed using all mitochondrial 13 PCGs to assess the phylogenetic position of *C. albus*. Our analysis, which included 17 additional avian species, placed *C. albus* (OR771717) in a strongly supported clade with *C. minor* within Charadrii (Figure 3). The tree further recovered the three major lineages of Charadriiformes (Charadrii, Scolopaci, and Lari).

## Discussion and conclusion

This study reports the first complete mitochondrial genome sequence of the snowy sheathbill (*C. albus*), a unique Antarctic bird belonging to the family Chionidae. The mitogenome is 19,116 bp in length and comprises the typical 13 PCGs, 22 tRNAs, two rRNAs, and a control region. Its overall gene organization and nucleotide composition are consistent with those of other avian mitochondrial genomes, indicating conserved features across Charadriiformes.

Notably, duplicated mitochondrial regions have been observed in the *C. albus* mitogenome. Similar duplications have been documented in other shorebirds, including *Calidris pugnax* (ruff), where duplications encompass *nad6*, several tRNAs, and a control region (Verkuil et al. 2010). However, such features are not universal across orders and are absent in some groups, such as Laridae (Yoon et al. 2015). In addition to Charadriiformes, mitogenomic duplications have also been reported in several Procellariiformes species (Abbott et al. 2005; Eda et al. 2010; Torres et al. 2019; Kim and Kim 2025). These findings indicate that duplications represent a recurring but lineage-specific mechanism in seabird mitochondrial genomes, with potential implications for regulatory functions and genomic plasticity. Furthermore, the read-mapping coverage remained continuous across the duplicated regions without abrupt drops (Supplementary Figure S1), supporting that these duplications are not assembly artifacts but are reliably represented in the sequencing data. Additionally, the *ND3* gene contained an extra nucleotide (Supplementary Fig. S2), a feature widely reported in birds and turtles (Mindell et al. 1998; Andreu-Sanchez et al. 2021) and also present in other Charadriiformes species such as *C. minor*, *Dromas ardeola*, *Rynchops niger*, and *Uria aalge* (Feng et al. 2020). This suggests that the feature is broadly distributed across avian mitogenomes, although its functional significance remains unclear.

The phylogenetic placement of *C. albus* as a strongly supported clade with *C. minor* supports the monophyly of Chionididae and its recognition as a distinct lineage within Charadriiformes. This placement is consistent with previous mitochondrial and multi-locus studies, and is further supported by recent comprehensive analyses integrating morphological and molecular data across the order (Černý and Natale 2022). Together, these findings highlight the conserved and unique features of the mitogenome of *C. albus* and provide insights into the mitochondrial evolution of Charadriiformes.

In conclusion, this study provides novel genetic resources for *C. albus* and members of the family Chionidae. This mitogenome provides a foundation for future research on avian molecular evolution, population genetics, and adaptation to polar environments. Additional mitogenomic data from related taxa are essential to better understand the evolutionary dynamics within Charadriiformes and the ecological role of sheathbills.

## Supplementary Material

Supplementary material.pdf

## Data Availability

The genome sequence data supporting the findings of this study are available in GenBank (https://www.ncbi.nlm.nih.gov/) under the accession no. OR771717. The associated BioProject, SRA, and Bio-Sample numbers are PRJNA1031807, SRR26685721, and SAMN37972956, respectively.
