# Supplementary material for: Complete mitochondrial genome of the snowy sheathbill (Chionis albus, Charadriiformes: chionidae) and its phylogenetic implications
Source: Mitochondrial DNA B Resour. 2026 Apr 18;11(5):630–5. doi: 10.1080/23802359.2026.2658389 (PMC13094258; doi:10.1080/23802359.2026.2658389)
Supplement: Supplementary material.pdf [file TMDN_A_2658389_SM5307.pdf]

Supplementary material

Complete mitochondrial genome of the snowy sheathbill (*Chionis albus*, Charadriiformes: Chionidae) and its phylogenetic implications

Jihee Kim*, Jong-U Kim, Jeong-Hoon Kim

Division of Life Sciences, Korea Polar Research Institute, Incheon, Korea

---

*Co-corresponding authors:

Jihee Kim, jiheeid@kopri.re.kr

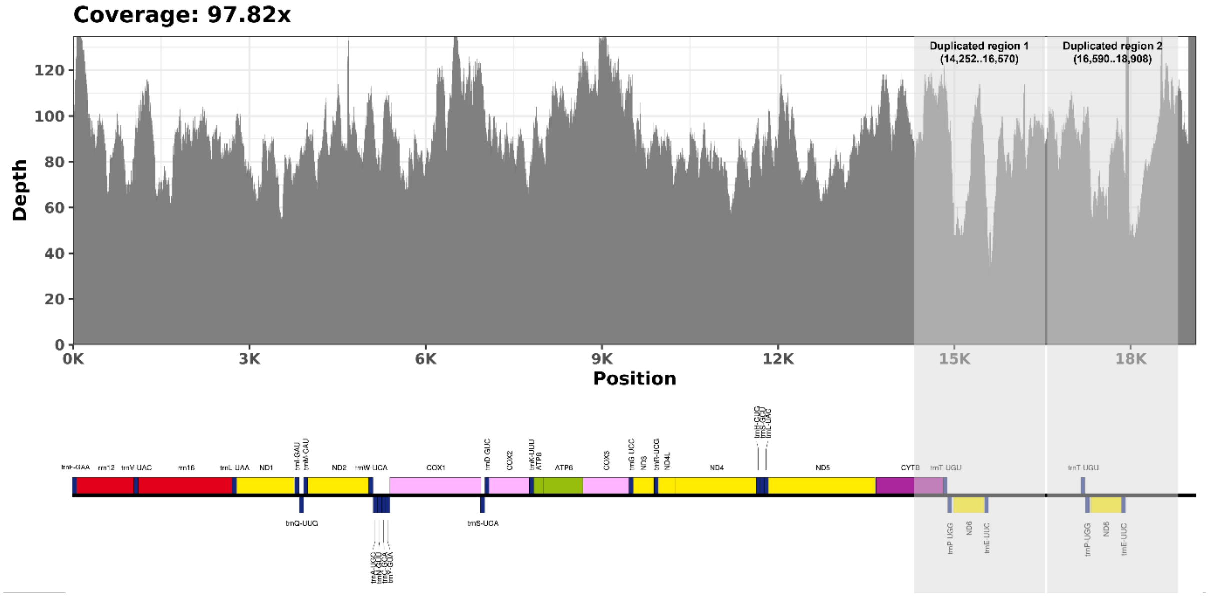

Supplementary Figure S1. Coverage depth plot of *Chionis albus* mitochondrial genome. The x-axis indicates the base position of the final assembly and the y-axis represents the number of reads aligned at each position.

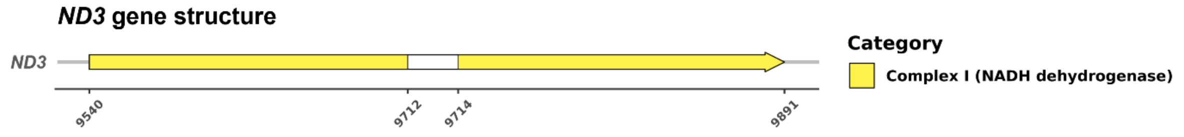

Supplementary Figure S2. Structure of the *ND3* gene of *Chionis albus* (OR771717), showing the extra nucleotide that induces a frameshift correction mechanism, a feature broadly conserved across birds.
